# 211 From Motion by Emotion to Well-Being: How Regular Physical Activity Enhances Life Satisfaction through Self–Control and Emotion Regulation

**DOI:** 10.1093/eurpub/ckae114.070

**Published:** 2024-09-26

**Authors:** Wiktor Potoczny, Lukasz Krzywoszanski, Radoslawa Herzog-Krzywoszanska

**Affiliations:** University Of The National Education Commission, Krakow, Krakow, Poland; University Of The National Education Commission, Krakow, Krakow, Poland; University Of The National Education Commission, Krakow, Krakow, Poland

## Abstract

**Purpose:**

Physical activity is crucial for maintaining good health and preventing chronic diseases. Regular participation in sports-related activities contributes to psychophysiological and social well-being. Researchers are investigating the psychological factors that may be responsible for this mechanism. Current research is one of the first to explore the mediating effects of self-control and emotion regulation on the relationship between exercise and life satisfaction. The main objective of this study was to examine the possible intermediate impact of self–control and two common forms of emotion regulation: cognitive reappraisal and expressive suppression on the relationship between involvement in regular physical activity and satisfaction with life.

**Methods:**

A total of 186 adults participated in the online survey, providing information about their regular participation in physical activity or training and completing questionnaires assessing life satisfaction, self-control, and two aspects of emotion regulation. Based on self-reported average minutes spent exercising per week over the past year, participants were classified into four levels of physical activity according to the World Health Organization guidelines on physical activity and sedentary behavior.

**Results:**

The results of the mediation analysis showed that dispositional self-control and cognitive reappraisal fully mediated the relationship between the levels of physical activity and life satisfaction. Expressive suppression, a second strategy of emotion regulation, did not mediate this relationship. This suggests that regular physical activity may increase levels of self-control and cognitive reappraisal, which in turn leads to greater life satisfaction.

**Conclusions:**

Regular physical activity and time spent on training support such forms of regulating one’s own emotions and actions which consequently may lead to greater life satisfaction. These findings underscore the importance of regular exercise in promoting psychological well-being through its beneficial effects on self-control and emotional regulation.

